# BAG3 in human tumors

**DOI:** 10.3389/fonc.2025.1725674

**Published:** 2025-11-24

**Authors:** Paola Manzo, Anna Lisa Cammarota, Jelena Dimitrov, Antonia Falco, Alessandra Rosati, Liberato Marzullo, Maria Caterina Turco, Vincenzo De Laurenzi, Gianluca Sala, Margot De Marco, Anna Basile

**Affiliations:** 1 Department of Medicine, Surgery and Dentistry “Scuola Medica Salernitana”, University of Salerno, Baronissi, Italy; 2 FIBROSYS srl, University of Salerno, Baronissi, Italy; 3 Center for Advanced Studies and Technology (CAST), “G. d’Annunzio” University of Chieti-Pescara, Chieti, Italy; 4 Department of Innovative Technologies in Medicine and Dentistry, “G. d’Annunzio” University of Chieti-Pescara, Chieti, Italy; 5 Department of Human, Philosophical and Educational Sciences (DISUFF), University of Salerno, Fisciano, Italy

**Keywords:** BAG3, tumor, cancer cell lines, human serum, biomarker

## Abstract

Previous studies identified BAG3 as a stress-induced protein with pro-survival functions in various tumors. Based on this assumption, we analyzed the expression and secretion of BAG3 in 24 cancer cell lines representing ten types of cancer and compared these results with samples from primary tumors. BAG3 was ubiquitously expressed and secreted by all cell lines. Serum levels of BAG3 were significantly elevated in patients with liver, pancreatic, and ovarian cancers versus healthy controls. Immunohistochemical analysis confirmed widespread high BAG3 expression across multiple tumor types, often correlating with tumor grade. These data support BAG3 as a key regulator of tumor survival and a promising biomarker and therapeutic target.

## Introduction

Bcl-2-associated athanogene 3 (BAG3) (1) is a multifunctional protein whose expression is induced by stressful stimuli, mainly through the activation of Heat Shock Factor (HSF) 1 (2), while is constitutive in human muscle cells, including cardiomyocytes (3–6), in brain and peripheral nervous system cells (6) and in some primary tumors (7–20). BAG3 interacts with the heat shock protein (Hsp)70 through its BAG domain, and with other partners through its WW domain, proline-rich (PXXP) repeat and IPV (Ile-Pro-Val) motifs, thereby regulating various intracellular pathways, including autophagy and mitophagy, apoptosis, mechanotransduction, excitation-contracting coupling, mitochondrial functions, cytoskeleton organization and motility, inflammasome modulation and structural stabilization of the sarcomere (6, 21–28). Stressful stimuli can induce the release of BAG3 through unconventional secretory pathways in certain cell types, such as cardiomyocytes and fibroblasts. BAG3 has indeed been found in the blood of patients with various cardiac or fibrotic diseases, including heart failure (29–32) and systemic sclerosis (33–35). Furthermore, BAG3 is detectable in the blood of patients affected by pancreatic adenocarcinoma (12, 15, 36).

The pro-survival activity of BAG3 (7, 8, 22–24) suggests that constitutive expression of this protein is a common characteristic of neoplastic cells, a hypothesis supported by analyses of multiple primary tumor types (7–20). In this study, BAG3 expression data integrated from numerous prior publications were reassessed, underscoring its widespread and robust association with tumor biology. Furthermore, the present work demonstrates that BAG3 is actively secreted by diverse human cancer cell lines, thereby extending previous knowledge that primarily focused on intracellular expression. Importantly, elevated serum levels of BAG3 were detected in patients with liver, pancreatic, and ovarian carcinomas compared to healthy controls, substantiating BAG3’s potential as a circulating biomarker reflective of tumor burden. These findings collectively reinforce the utility of BAG3 not only as a tissue-level marker but also as a secreted protein detectable in blood, opening new avenues for non-invasive cancer diagnostics and targeted therapeutic strategies.

## Methods

### Cell cultures

The human pancreatic cancer cell line HPAAPC (Cytion, Freiburg, Germany) was grown in a 1:1 mix of DMEM and Ham’s F12 medium with 5% FBS, 1% penicillin/streptomycin (P/S), 0.5 mM sodium pyruvate, 0.002 mg/mL insulin, 0.005 mg/mL transferrin, 40 ng/mL hydrocortisone, and 10 ng/mL mouse epidermal growth factor. The anaplastic thyroid carcinoma cell line 8505C (ECACC, Salisbury, UK) was cultured in EMEM with 10% FBS, 2 mM glutamine, and 1% non-essential amino acids (NEAA). Fibrosarcoma HT-1080 cells (ATCC) were grown in EMEM with 10% FBS. Liver cancer lines SK-Hep-1 and HepG2 (ATCC) were cultured in EMEM with 10% FBS and 1% P/S, while SNU-475, SNU-423, and SNU-387 (ATCC) were grown in RPMI-1640 with 10% FBS and 1% P/S. The gastric adenocarcinoma MKN-45 line (DSMZ, Germany) was cultured in RPMI-1640 with 20% FBS and 1% P/S. Head and neck cancer cell lines A-253, Detroit 562, SCC-9, and FaDu (ATCC) were cultured in different media: A-253 in McCoy’s 5A with 10% FBS and 1% P/S; Detroit 562 and FaDu in EMEM with 10% FBS and 1% P/S; SCC-9 in 1:1 DMEM and Ham’s F12 with 10% FBS, 1% P/S, 0.5 mM sodium pyruvate, and 400 ng/mL hydrocortisone. Melanoma lines A375, SK-Mel-24, SK-Mel-28, C8161, and UACC25 (ATCC) were cultured as follows: SK-Mel-24 and SK-Mel-28 in EMEM with 15% FBS and 1% P/S; C8161 in 1:1 DMEM and Ham’s F12 with 10% FBS; UACC25 in RPMI-1640 with 10% FBS and 1% P/S. Ovarian cancer lines PEA-1 and PEA-2 (ECACC) were grown in RPMI-1640 with 10% FBS, 2 mM glutamine, 2 mM sodium pyruvate, and 1% P/S. Breast cancer lines MCF-7 and MDA-MB-231 (ATCC) were cultured with MCF-7 in EMEM plus 10% FBS, 2 mM glutamine, 1% NEAA, and 1% P/S, and MDA-MB-231 in DMEM with 10% FBS and 1% P/S. All cell lines were maintained at 37°C in a humidified atmosphere containing 5% CO_2_.

### Isolation of cell culture supernatants

Tumor cells were plated in complete medium at a density of 1x10^6^ cells/ml. The day after, were washed twice with 1X PBS and incubated for 16 hours in serum-free DMEM. The conditioned medium was collected and subjected to sequential centrifugation steps at 4°C to eliminate dead cells and cellular debris. The cleared supernatant was precipitated overnight at -20°C using cold acetone using a volume ratio ratio 1:3. After incubation, the samples were centrifuged again at 10, 000 x g for 30 minutes, and the supernatant was discarded. The protein pellet was then analyzed by Western Blot.

### Western blot

Intracellular proteins were obtained by using the TNT buffer (20mM HEPES (pH 7.5), 150mM NaCl, 0.1% Triton) containing a protease inhibitor cocktail (Sigma‐Aldrich), and subjected to 3 cycles of freezing/thawing. Lysates were then centrifuged for 20 min at 15, 000g, and the cleared supernatants were stored at -80°C. Protein concentration was determined by Bradford assay (Bio‐Rad), and 20 μg of total protein were separated on 10% SDS‐PAGE gels and electrophoretically transferred onto a nitrocellulose membrane. Nitrocellulose blots were blocked with 10% nonfat dry milk in TBST buffer (20mM Tris‐HCl at pH 7.4, 500mM NaCl, and 0.01% Tween), and incubated with primary antibodies in TBST containing 5% nonfat dry milk overnight at 4°C. An anti-BAG3 polyclonal antibody obtained by immunizing rabbits with the full- lenght recombinant BAG3 protein, anti-GAPDH monoclonal antibody (sc-32, 233, Santa Cruz Biotechnology), anti-Calregulin polyclonal antibody (sc-11398, Santa Cruz Biotechnology), anti-beta actin monoclonal antibody (sc-47778, Santa Cruz Biotechnology), anti-beta Tubulin monoclonal antibody (sc-166729, Santa Cruz Biotechnology), and anti-TRAP-1 monoclonal antibody (sc-73604, Santa Cruz Biotechnology), were used at a 1:5000 dilution. Immunoreactivity was detected using an ImageQuant ™ LAS 4000 (GE Healthcare).

### Serum samples

Aliquots of serum samples were purchased at BIOIVT (West Sussex, United Kingdom) or at ReproCELL USA, Inc. (Maryland, USA) and stored at -80°C. The data on sera from healthy subjects analyzed in this study have been previously published (34).

### BAG3 determination by the ELISA test

The BAG3 protein content in serum was measured using an enzyme-linked immunosorbent assay (ELISA). 96-well microplates (MediSorp™, cat. no. 467320, Thermo Scientific, Waltham, MA, USA) were coated with a proprietary monoclonal anti-BAG3 coating mAb and then blocked for non-specific binding sites. BAG3 standard protein or serum samples were then added to the wells. BAG3 content in sera samples was then determined using a second recombinant anti-BAG3 HRP-conjugated antibody as previously described (32).

### Statistical analysis

Results were analyzed by GraphPad Prism software version 8.0.1 (Boston, MA, USA) and G*power software version 3.1.9.4. For variables non-normally distributed, p-values were assessed by a non-parametric Mann-Whitney U test to compare BAG3 serum level in individual patient populations with different carcinomas to healthy subjects. The statistical power analysis was conducted assuming these input parameters: statistical test: Mann-Whitney test; tail(s): one; parent distribution: min ARE; alpha error prob.: 0.05; effect size: calculated from the mean and standard deviation values obtained from the ELISA assay.

## Results

### BAG3 presence in the media from tumor cells and in patients’ serum

**Figure 1:**
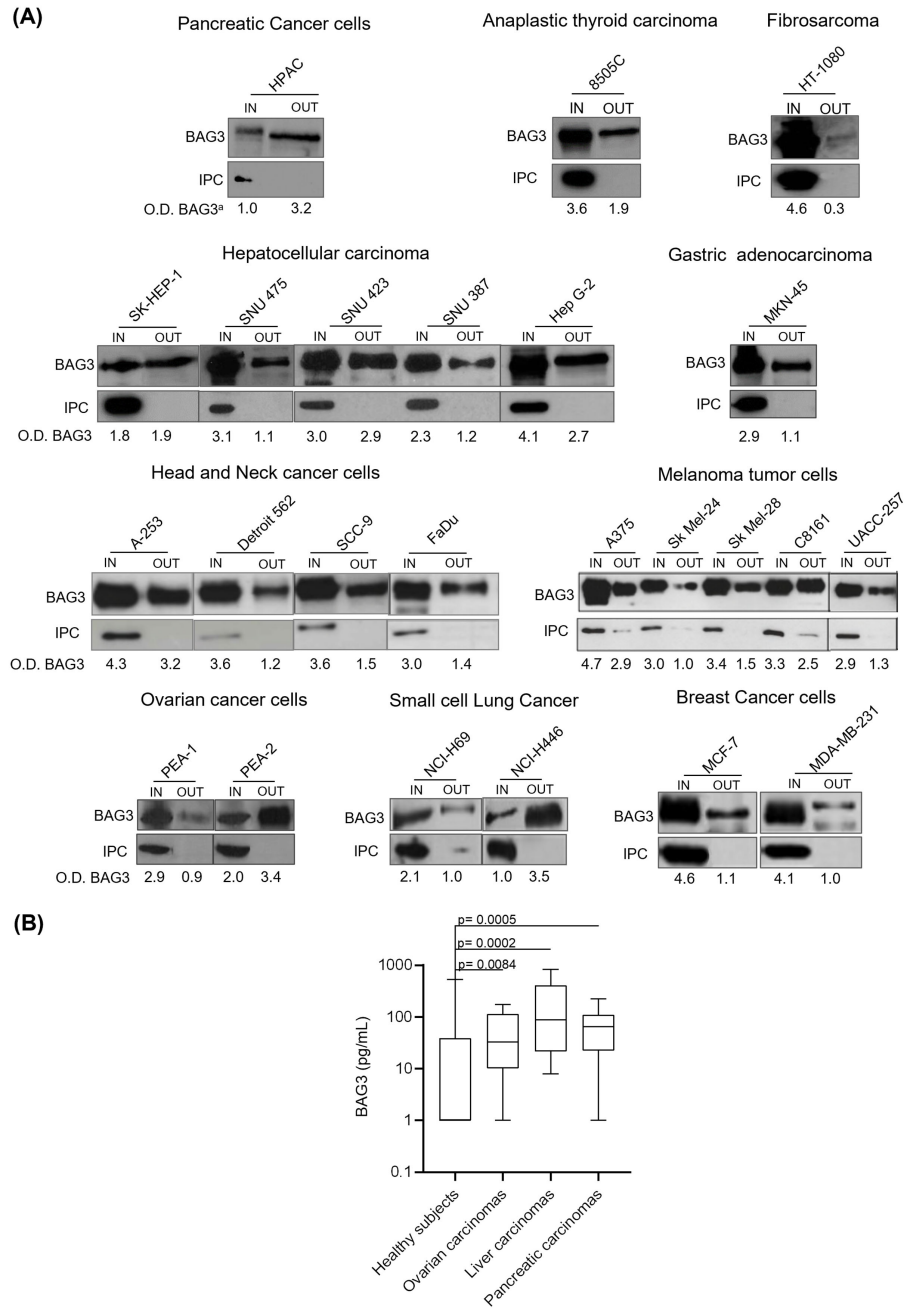
Detection of BAG3 in conditioned media from different cell types and in serum samples from patients with oncological diseases. **(A)** Human tumor cells were seeded at a density of 1x10^6^ cells/mL. Twenty-four hours later, the cells were incubated for 16 hours in serum-free DMEM at 37°C in a 5% CO2 atmosphere. Total protein extracts from the cells (IN) and proteins from supernatants (OUT) were analyzed by Western blotting using a proprietary anti-BAG3 polyclonal antibody. Antibodies against GAPDH, β-actin, β-tubulin, calregulin, and TRAP1 were used as intracellular protein controls (IPC). O.D. BAG3^a^: optical density of BAG3 protein normalized to cell number. **(B)** Serum from patients with ovarian carcinoma (N=10), pancreatic carcinoma (N=10), and liver carcinoma (N=10) was tested for BAG3 protein levels through an ELISA assay. The graph shows the BAG3 values of patients’ sera compared to healthy subjects (N=191).

The results shown in **Figure 1A** demonstrate that the BAG3 protein was found in both cell extracts (IN) and culture supernatants (OUT) from a variety of human cancer cell lines, indicating active secretion into the extracellular environment. In particular, Western blot analyses revealed that pancreatic cancer cell lines displayed BAG3 signals in both cell lysates and supernatants, confirming previous findings that indicated BAG3 secretion by pancreatic ductal adenocarcinoma cells (37). Other tumor cell lines, including anaplastic thyroid carcinoma (8505C), fibrosarcoma (HT-1080), hepatocellular carcinoma (SK-Hep-1, SNU-475, SNU-423, SNU-387, HepG2), gastric adenocarcinoma (MKN-45), head and neck cancer (A-253, Detroit 562, SCC-9, FaDu), melanoma (A375, SK-Mel-24, SK-Mel-28, C8161, UACC257), ovarian cancer (PEA-1, PEA-2), small cell lung cancer (NCI-H69, NCI-H446), and breast cancer (MCF-7, MDA-MB-231), also displayed intracellular BAG3 expression and BAG3 release, although secretion levels varied among different lines.

Additionally, we measured BAG3 protein levels in serum samples from patients with various carcinomas (**Figure 1B**). The median BAG3 concentrations in these patients were higher than in healthy subjects. Specifically, median values were 88 pg/ml in liver carcinoma patients (p value vs healthy subjects = 0.0002; power (1-𝛽) = 0.86), 65 pg/ml in pancreatic carcinoma patients (p value vs healthy subjects = 0.0005; power (1-𝛽) = 0.65), and 33 pg/ml in ovarian carcinoma patients (p value vs healthy subjects = 0.0084; power (1-𝛽) = 0.35), while the median level in healthy subjects was under 15 pg/ml, which corresponds to the assay’s lower limit of detection. No significant association was observed between BAG3 levels and patients’ age or sex within the studied populations. These baseline characteristics data are summarized in **Table 1**.

**Table 1:** Healthy subjects and patients’ characteristics.

Healthy subjects			Patients		
			Liver cancer	Ovarian cancer	Pancreatic cancer
Total	N	191	10	10	10
Age	Median (IQR)	49.0 (35.0 to 59.0)	62.5 (57.2 to 70.0)	55.5 (37.5 to 59.2)	54.0 (52.2 to 63.0)
Gender %	F	59.2	10.0	100.0	40.0
	M	40.8	90.0	0.0	60.0

### High BAG3 expression across a spectrum of human cancers

**Figure 2:**
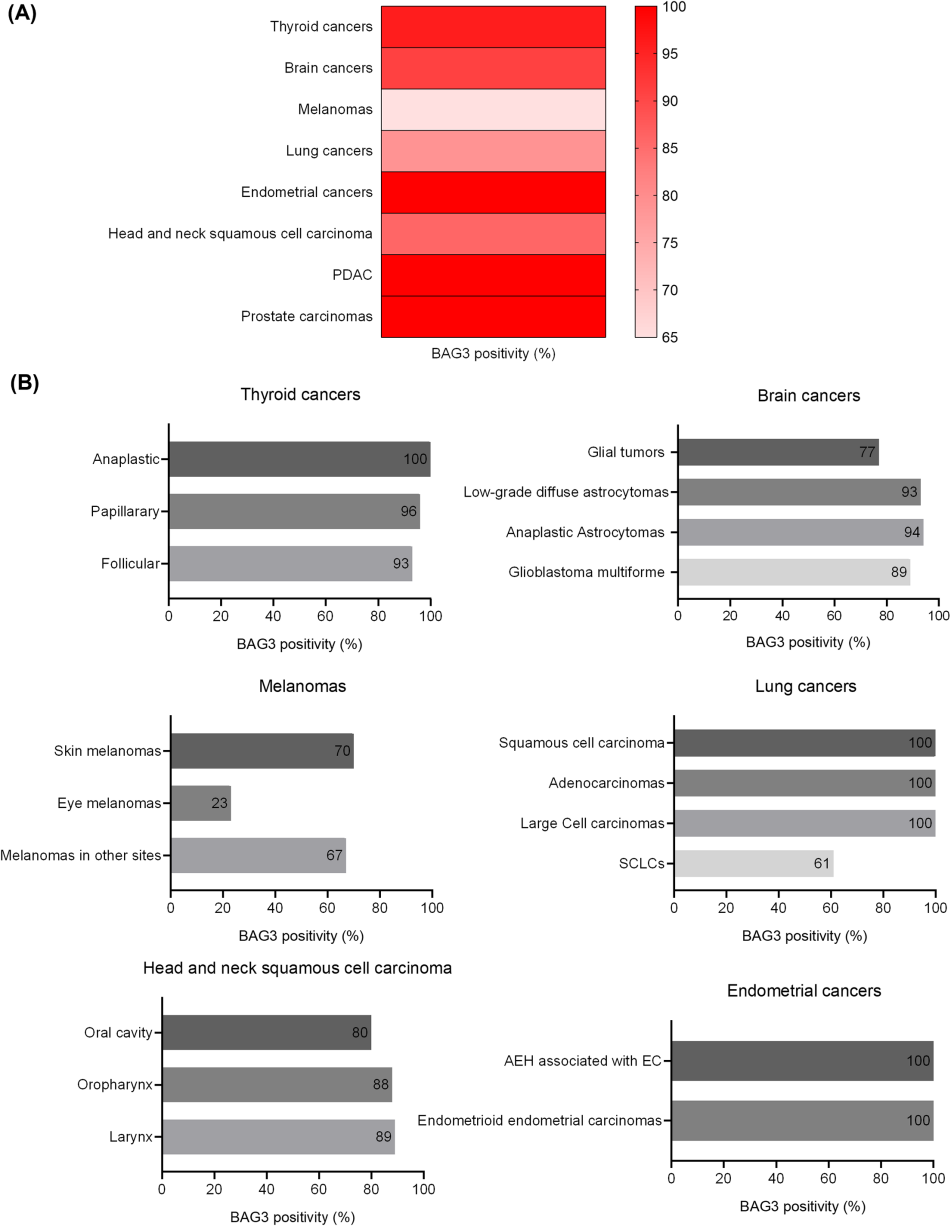
BAG3 positivity rates across human cancers and specific subtypes. The figure illustrates the percentage of BAG3-positive cases identified across a panel of human malignancies. The data show the overall positivity rate for each primary cancer type **(A)** and the specific rates for their respective histological subtypes **(B)**.

BAG3 protein expression was previously analyzed in our lab in various human tumors, showing high positivity rates consistent with its role as an anti-apoptotic factor. As summarized in **Figure 2** and in **Supplementary Table 1**, high levels of cytoplasmic BAG3 positivity were detected in various malignancies, often exceeding 90%. Specifically, endometrial tumors, PDAC (pancreatic ductal adenocarcinoma), and prostate carcinomas all showed positivity in 100% of the 515 cases analyzed. Equally high rates were observed in thyroid tumors (96% of 56 cases) and brain tumors (91% of 151 cases). While thyroid tumors showed consistently high BAG3 expression in all subtypes (papillary 96%, follicular 93%, and anaplastic 100%), expression in brain tumors varied by grade, with grade I glial tumors showing 77%, while grade II and III astrocytomas and glioblastoma multiforme all showed values above 89%. In addition, lung tumors (79% of 66 cases) showed 100% positivity in squamous cell carcinoma, adenocarcinoma, and large cell carcinoma, dropping to 61% in SCLC (Small Cell Lung Cancer). Head and neck squamous cell carcinoma (HNSCC) also showed high rates (86% overall, with oral cavity 80%, oropharynx 88%, and larynx 89%). The lowest overall positivity rate was found in melanomas (65% of 165 cases), where positivity was high in cutaneous melanomas (70%) and melanomas at other sites (67%), but significantly lower in ocular melanomas (23%). These results, derived from our previous publications (11, 13, 15, 17, 20, 38–42), underscore the widespread and elevated expression of the anti-apoptotic BAG3 protein in diverse cancers.

## Discussion

Our data reveal that BAG3 is both expressed and secreted by 24 tumor cell lines from diverse origins, highlighting the need for further investigation into its role in tumor growth *in vitro*. These findings confirm that BAG3’s function goes beyond its well-known intracellular activities to include secretion by cancer cells, which aligns with its involvement in regulating survival, proliferation, and intercellular signaling within the tumor microenvironment. Moreover, elevated serum BAG3 levels in carcinoma patients compared to healthy controls further support its potential as a biomarker for disease presence and activity.

This study reinforces and broadens existing evidence that the anti-apoptotic protein BAG3 exhibits high expression across a broad range of human malignancies, frequently exceeding 90% positivity. Specific tumor types such as endometrial, pancreatic ductal adenocarcinoma, and prostate cancers show near-universal BAG3 expression. Significant expression is also observed in certain thyroid, brain, lung, and head and neck cancer subtypes, accompanied by notable intertumoral heterogeneity, especially in melanomas and small cell lung cancers. These data substantiate BAG3’s function as a pivotal survival factor in tumor biology. Notably, aggressive tumor subtypes, including anaplastic thyroid carcinoma and glioblastoma—which are characterized by therapy resistance and poor prognosis—demonstrate elevated BAG3 positivity, suggesting BAG3-driven mechanisms contribute to their aggressive phenotype. Lower BAG3 levels in ocular melanomas and small cell lung cancer may indicate distinct cellular origins or regulatory processes. While BAG3 positivity correlates with tumor grade in some contexts, BAG3-negative tumors likely depend on alternative molecular alterations or signaling inputs from the microenvironment to maintain viability.

Nevertheless, the widespread secretion of BAG3 by tumors emphasizes its critical role in shaping the tumor microenvironment. Indeed, extracellular BAG3, through interaction with the IFITM2 receptor on macrophages and fibroblasts, fosters a pro-tumorigenic milieu that supports tumor growth, invasion, and immune evasion (37, 43, 44). Therapeutically, targeting extracellular BAG3 with monoclonal neutralizing antibodies in murine pancreatic adenocarcinoma models reduced fibrosis and macrophage infiltration, resulting in inhibited tumor progression (37, 45). Moreover, combining extracellular BAG3 blockade with immune checkpoint inhibitors (such as anti-SIRP-α or anti-PD-1 antibodies) (46, 47) yields synergistic enhancement of anti-tumor immune responses beyond immune checkpoint inhibition alone. These findings highlight BAG3’s potential as both a biomarker of tumor aggressiveness and a promising therapeutic target to disrupt malignant cell-microenvironment crosstalk.

The limited sample sizes for each tumor type, especially for ovarian carcinoma, where the statistical power was low, restrict the strength of our conclusions regarding serum BAG3 levels. Notably, ovarian carcinoma cells, particularly the chemoresistant line (PEA-2), display a distinct pattern of BAG3 secretion compared to other cancers. While our current study did not explicitly investigate the influence of metabolic changes on BAG3 secretion, the established reliance of ovarian cancer on oxidative metabolism suggests a potential metabolic link that warrants further exploration. Additionally, the cross-sectional nature of the serum analysis and the proximity of healthy subject BAG3 levels to the assay’s detection limit suggest the need for larger, longitudinal studies to validate BAG3’s utility as a reliable circulating biomarker in oncology.

Collectively, BAG3 expression and secretion are characteristic of many neoplasms, and understanding their precise functional roles in diverse tumor types can provide insights into tumor survival mechanisms and inform the development of innovative therapeutic strategies.

## Data Availability

The raw data supporting the conclusions of this article will be made available by the authors, without undue reservation.
