# Supplementary material for: BAG3 in human tumors
Source: Front Oncol. 2025 Nov 24;15:1725674. doi: 10.3389/fonc.2025.1725674 (PMC12682678; doi:10.3389/fonc.2025.1725674)
Supplement: Supplementary file 1 [file Table1.docx]

**Supplementary Table 1: BAG3 positivity rates in human tumors.**

| **Tumor Types** | **Cases Analyzed (n)** | **Positivity**  **%** | **Sub-Type** | **Positivity**  **%** | **Cases Analyzed (n)** |
| --- | --- | --- | --- | --- | --- |
| Thyroid cancers | 56 | 96 | Follicular | 93 | 15 |
|  |  |  | Papillarary | 96 | 28 |
|  |  |  | Anaplastic | 100 | 13 |
| Brain cancers | 151 | 91 | Glial tumors (grade I) | 77 | 13 |
|  |  |  | Low-grade diffuse astrocytomas (grade II) | 93 | 85 |
|  |  |  | Anaplastic Astrocytomas (grade III) | 94 | 17 |
|  |  |  | Glioblastoma multiforme (grade IV) | 89 | 36 |
| Melanomas | 165 | 65 | Skin melanomas | 70 | 100 |
|  |  |  | Eye melanomas | 23 | 13 |
|  |  |  | Melanomas in other sites | 67 | 52 |
| Lung cancers | 66 | 79 | Squamous cell carcinoma | 100 | 13 |
|  |  |  | Adenocarcinomas | 100 | 13 |
|  |  |  | Large cell carcinomas | 100 | 4 |
|  |  |  | SCLCs | 61 | 36 |
| Endometrial cancers | 114 | 100 | Endometrioid endometrial carcinomas | 100 | 14 |
|  |  |  | AEH associated with endometrial cancers | 100 | 100 |
| Head and neck squamous cell carcinoma | 104 | 86 | Oral cavity | 80 | 30 |
|  |  |  | Oropharynx | 88 | 8 |
|  |  |  | Larynx | 89 | 66 |
| PDAC | 346 | 100 |  |  |  |
| Prostate carcinomas | 55 | 100 |  |  |  |
